# Climate change and health in international medical education – a narrative review

**DOI:** 10.3205/zma001619

**Published:** 2023-05-15

**Authors:** Rebecca Boekels, Christoph Nikendei, Emma Roether, Hans-Christoph Friederich, Till Johannes Bugaj

**Affiliations:** 1 Heidelberg University Hospital, Department of General Internal Medicine and Psychosomatics, Heidelberg, Germany

**Keywords:** global warming, medical education, health education, environmental health, climate change

## Abstract

**Objective:**

Climate change is a key threat to human health worldwide. Accordingly, medical education should prepare future physicians for climate-associated hazards and corresponding professional challenges. Currently, this is not yet implemented across the board. The aim of this review is to present (I) the knowledge and (II) the attitudes of medical students and physicians towards climate change and (III) the expectations of medical education as formulated by medical students. In addition, the available literature will be used to look at (IV) global teaching activities, (V) international learning goals and learning goal catalogues, and (VI) applied teaching methods and formats. This review should simplify and, considering the urgency of the topic, accelerate the design of future teaching activities.

**Methodology:**

The paper is based on a selective literature search supplemented by a topic-guided internet search.

**Results:**

Knowledge about the causes and concrete health consequences of climate change seems to be incomplete. The majority of medical students consider human health to be at risk from climate change and the health sector to be inadequately prepared. A majority of surveyed medical students would like to see teaching about climate change. It is evident that internationally, teaching projects on climate change and climate health, as well as topic-specific learning objectives and learning goal catalogues, have been developed and integrated into medical education.

**Conclusion:**

There is a need for and acceptance of teaching climate change in the medical curriculum. This literature review can assist in the development and implementation of new teaching formats.

## Zusammenfassung

**Zielsetzung:**

Der Klimawandel stellt eine zentrale Bedrohung für die Gesundheit der Menschen weltweit dar. Entsprechend sollte die medizinische Ausbildung zukünftige Ärzt*innen auf klimaassoziierte Gefahren und entsprechende berufliche Herausforderungen vorbereiten. Aktuell wird dies noch nicht flächendeckend umgesetzt. Ziel dieser Übersichtsarbeit ist es, (I) das Wissen und (II) die Haltungen von Medizinstudierenden und Ärzt*innen zum Klimawandel sowie (III) die von den Medizinstudierenden formulierten Erwartungen an die medizinische Lehre darzustellen. Darüber hinaus wird anhand der verfügbaren Literatur ein Blick auf (IV) globale Lehraktivitäten gelenkt, (V) internationale Lernziele und Lernzielkataloge werden dargestellt sowie (VI) angewandte Lehrmethoden und -formate beleuchtet. Hierdurch soll die Gestaltung künftiger Lehrveranstaltung vereinfacht und – in Anbetracht der Dringlichkeit des Themas – beschleunigt werden.

**Methodik:**

Dem Beitrag liegt eine selektive Literaturrecherche, ergänzt durch eine themengeleitete Internetsuche, zugrunde.

**Ergebnisse:**

Das Wissen um die Ursachen und konkrete gesundheitliche Folgen des Klimawandels scheint unvollständig zu sein. Mehrheitlich betrachten Medizinstudierende die menschliche Gesundheit als durch den Klimawandel gefährdet und den Gesundheitssektor als inadäquat vorbereitet. Eine Mehrheit befragter Medizinstudierender wünscht Lehre zum Themenkomplex Klimawandel. Es zeigt sich, dass international Lehrprojekte zu Klimawandel und Klimagesundheit sowie themenspezifische Lernziele und Lernzielkataloge entwickelt und in die medizinische Ausbildung integriert wurden.

**Schlussfolgerung:**

Es besteht der Bedarf und eine Akzeptanz für Lehre zum Klimawandel im medizinischen Curriculum. Die vorliegende Literaturübersicht kann bei der Entwicklung und Implementierung neuer Lehrformate unterstützen.

## 1. Introduction

Climate change threatens the health, well-being, and lives of countless people [1], [2]. The effects of global warming on human health are manifold; on the one hand, heat waves, extreme weather events, changes in air quality and alterations of ecosystems lead to direct negative health impacts [3], while on the other hand, climate change indirectly affects human health through drinking water shortages, flight movements and higher (also violent) conflict potential [3]. In this context, the poorer and vulnerable parts of humanity are particularly affected, as they are already at risk from malnutrition, extreme weather events or sea-level rise [3].

Health systems worldwide are being confronted with the resulting health problems, which is why there is an urgent need and responsibility for medical professionals to educate themselves in this regard. Their responsibility includes at least four levels: as physicians, they must be able to recognize, prevent and treat so-called climate-sensitive and climate-induced diseases [4]; as scientists, they must also be able to collect, process and interpret the relevant medical data. Likewise, it is necessary to limit greenhouse gas (GHG) emissions on an institutional level (mitigation) and to mitigate the consequences of climate change through adaptation measures (adaptation) [3]. The central role of physicians in supporting a societal transformation represents another area; specifically, the trust placed in them, or their social status [5], empowers them to become leaders in addressing climate change [5], to serve as societal role models and to help initiate change in business, politics, and education [6]. The medical workforce [4] must be adequately prepared for these roles. Teaching oriented to the requirements of climate change is therefore indispensable [7]. Nevertheless, teaching on climate change is still far from being established at every medical school. To implement much needed climate health teaching within one's faculty, the following points should be considered:

- Medical students' knowledge and attitudes about climate change should be known, as well as their expectations in this regard, to tailor teaching to the actual needs of the target group.
- In addition, knowledge about pilot projects, already established courses or published learning objective catalogues is helpful to benefit from this preliminary work.
- Furthermore, adequate teaching methods adapted to the content should be used.

All these points have already been addressed in international publications, the results of which are the focus of this review.

## 2. Methods

### 2.1. Objective

The objective of this review is to present the state of international medical education on climate change and health. For this purpose, findings on prior knowledge, attitudes and expectations of medical students on climate change and climate science were collected. Current teaching activities were considered, and possible content and design options for specific courses were identified.

#### 2.2. Methodological approach

**Figure 1:**
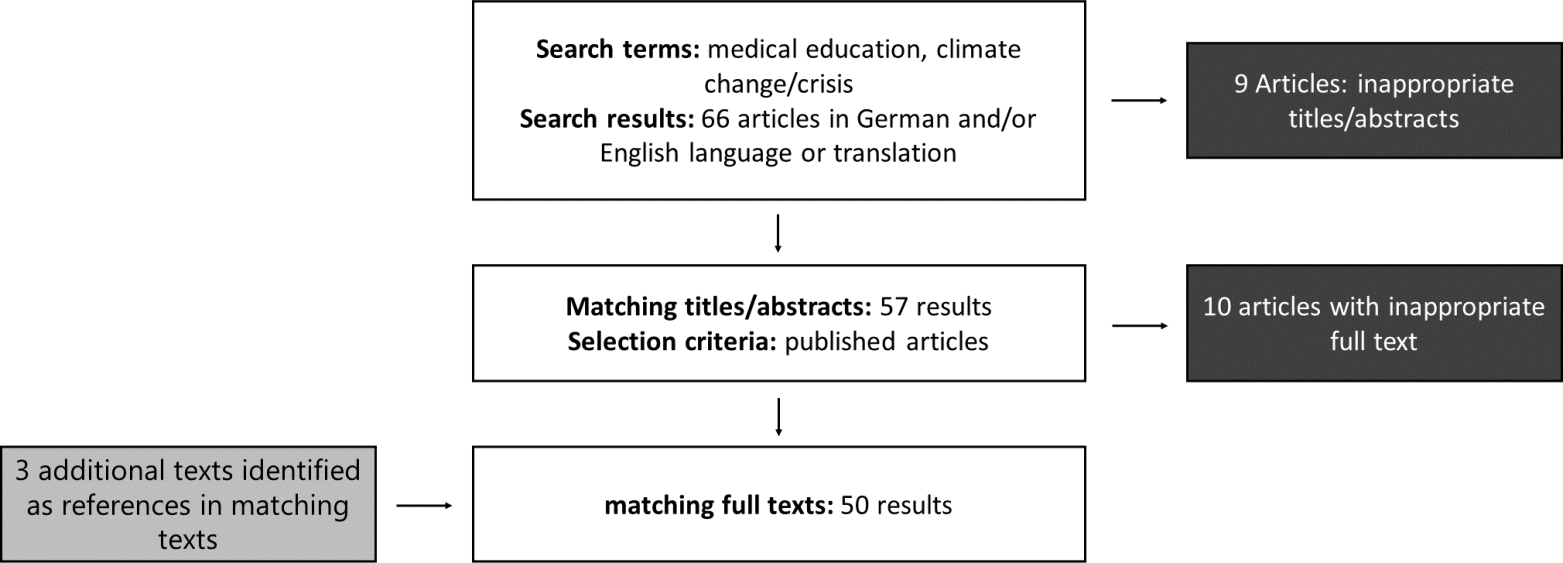
Search strategy

A three-step approach was followed for the literature review. First, areas were identified on which to focus the research, namely, (I) knowledge, (II) attitudes of medical students and physicians and related disciplines towards climate change, (III) expectations of medical students regarding medical teaching, (IV) a global representation of existing teaching activities, (V) formulated learning objectives, and (VI) applied methods and formats in teaching climate-related content. The search for relevant publications was conducted in the PubMed and Google Scholar databases in April 2021. The search strategy is presented in figure 1 (Fig. 1). Following this orienting search, a second, more in-depth search was conducted that included references of appropriate articles. The focus here was on publications that addressed the teaching of climate change content in human medical education. Finally, the identified articles were reviewed and elaborated upon, based on which this narrative review was written.

## 3. Target group analysis: knowledge and attitudes towards climate change and expectations towards climate teaching

### 3.1. Knowledge of medical students on climate change and sources of information

In a study of German medical students in their internship year, 72% of the respondents affirmed the statement that climate change is almost entirely man-made [8], which represents the current scientific consensus regarding climate research [9]; however, the reasons for the disagreement of the remaining 28% were not further investigated [8]. In an interprofessional study from the United States, Yale University medical students, among others, were surveyed about their knowledge and attitudes towards climate change. Fifty-seven percent of the participants – consisting of medical students, nursing students, and physician assistants in training – underestimated the amount of greenhouse gases emitted by the U.S. health care sector [10]. A Chinese study surveyed students from several professions and assessed their knowledge of climate change [11]. The questions used included human responsibility for climate change, the increase in CO_2_ concentration in the atmosphere, and the increase in the Earth's temperature. The questions were answered correctly by 60% of the students. It was further revealed that a majority of the respondents (79%) considered humans to be mainly responsible for climate change [11]. Similarly, three-quarters of the medical students surveyed stated that there had been a global increase in CO_2_ in the atmosphere within the last 250 years [11]. Individual direct health consequences of climate change, for example, poorer air quality or heat stress, were recognized by 84-94% of the respondents, while only a few identified malnutrition and mental illness as possible indirect consequences of climate change [11]. Another article from China shed light on the sources of information used by medical students. The internet was most frequently cited as a source of information on the climate crisis, followed by television and radio [12]. Teaching staff was the third most frequently cited source of information [12]. Similarly, a survey of Ethiopian medical students, as well as students of other health disciplines, showed that these students used electronic mass media as their main source of information on climate change [13]. Nevertheless, almost 80% of Chinese medical students, as well as the majority of Ethiopian students, stated that they lacked necessary knowledge for dealing with climate change health risks [12], [13]. This lack was attributed to their education by 90% of Ethiopian students. For example, relevant knowledge includes knowledge of vulnerable groups. Although Chinese medical students often recognized a large proportion of relevant at-risk groups, such as senior citizens, children, people working outdoors, people with health limitations, and people living in certain geographic regions, they less often identified people of lower socioeconomic status as vulnerable populations [12].

#### 3.2. Knowledge of physicians on climate change and sources of information

Surveys of the current state of knowledge on climate change among already licenced physicians is also informative to derive which teaching approaches medical students – as tomorrow's physicians – could benefit from. For example, in 2015 and 2016, the American Thoracic Society (ATS) surveyed its international members regarding their knowledge of the links between climate change and health. In 2015, 89% of physicians agreed that global temperatures had risen in the past, would continue to rise, and would change the world's climate. Sixty-eight percent saw humans as the cause of these changes [14]. One year later, 96% of the respondents thought that the global temperature increase was real; however, this time, humans were blamed by 70% of the respondents [15]. In addition to simply acknowledging climate change, broader knowledge about its causes and effects is very relevant to physicians. Only 54% of the respondents in 2015 described themselves as fairly or very knowledgeable about climate change. Six percent of the respondents said they were not informed at all [14]. This lack of knowledge was also widely perceived as a barrier in communicating with patient; in addition to lack of time, 45% of physicians cited as a problem that they did not know how to discuss the health impacts of climate change with their patients [15]. Reports from the Intergovernmental Panel on Climate Change (IPCC) were named by 40% of the physicians as a trustworthy source of information [14]; one year later, approximately half of the respondents made this same claim [15].

One group that is particularly confronted with the health impacts of climate change is rural physicians. In 2014, a survey of rural physicians was conducted in Australia. Among them, 71% considered climate change to be real, and 66% attributed such change to greenhouse gas emissions [16].

#### 3.3. Attitudes of medical students towards climate change

The majority of German medical students in a 2021 study considered themselves to be role models regarding climate change, while less than half of the respondents reported having either a particular social responsibility or informative or educational role [8]. Among Yale University medical students, 93% reported being concerned about the health impacts of climate change [10]. A majority (88%) of the respondents felt a responsibility to work in a resource- and environmentally friendly manner [10]. Sixty percent of the medical students felt it was important to understand climate change to help patients. Among female students, agreement with this corresponding statement was higher. A majority (almost 70%) felt that it is part of a physician's duties to inform patients and the public about climate change [10]. This finding contrasts with the results of the German study, in which only 40% of students saw the medical profession has having an educational role [8]. Another survey was conducted nationwide among medical students and public health and nursing students in China; more than 80% of the students’ surveyed by Yang et al. expected negative effects of climate change on human health [11]. Sixty-five percent of the respondents thought such changes were controllable [11]. A large majority, i.e., 96% and 94%, agreed that climate change would have a negative impact on both China and the world, respectively [11]. Another study of Chinese medical students showed that approximately half of the respondents did not think the health sector was adequately prepared for climate change, although 90% of participants considered addressing the health consequences of climate change to be their responsibility [12].

#### 3.4. Attitudes of physicians towards climate change

Since the number of studies focusing on medical students is small, the attitudes of already licenced physicians will be considered comparatively. In the 2015 and 2016 ATS studies, medical students from various disciplines were surveyed [14], [15]. Compared to Yale University medical students, these students were 65% more likely to express that climate change was relevant to patient care and was already affecting the health of their patients [14]. Nearly 80% of the physician respondents felt a responsibility to teach their patients about the health impacts of climate change. An equal number of respondents agreed that this responsibility extended to the general population [14], [15]. Among rural physicians surveyed by Purcell et al. in 2014, more physicians saw themselves in an educational role for general health education. Significantly fewer respondents (65%) also considered educating patients about climate change and health to be important. This finding correlated with age, as it was mainly physicians under 55 years of age who perceived such education to be their task [16].

#### 3.5. Attitude of medical students towards the integration of planetary health teaching content in the curriculum

In most of the identified studies, most medical students showed positive attitudes towards the introduction of climate change coursework and content. In a study based in the United States, Ryan et al. showed that nearly 60% of medical students surveyed wanted the topic of climate change to be integrated into their curriculum [10]. However, 30% felt that their studies were already sufficiently time-consuming without this new content [10]. The teaching of this topic was explicitly rejected by 17% of medical students [10]. A similar picture was shown by one of the two studies conducted at Chinese universities already mentioned herein; the most frequently cited reason for a lack of necessary knowledge on climate change was the lack of a theoretical superstructure, and the second most frequent reason was a lack of teaching [12]. The vast majority of students – between 70-80%, depending on the course of study – wanted content on climate change to be added to their curriculum [12].

#### 3.6. Requirements perceived by students in the teaching of nonmedical professions

Due to the very limited number of studies on the desire to address climate change in the human medical curriculum, it is also recommended to look at studies in other health professions. In 2014, nursing trainees in four European countries were asked whether they wanted climate change events to be integrated into their curriculum. German nursing trainees were significantly more likely to agree than those from the United Kingdom [17]. In 2016, it was also shown in various Arab countries that nursing students have a positive attitude towards the topics of climate change and sustainability [18].

## 4. Teaching events and appropriate learning objectives worldwide

###  4.1. Status quo of climate change teaching worldwide

In many places, courses that teach climate change have already been piloted or permanently implemented in medical education. A 2019 survey of members of the International Federation of Medical Students Associations (IFSMA) showed that 73 of 107 countries had integrated climate change teaching into their national curricula [19]. Four months later, 2817 medical schools in 108 countries were surveyed to determine whether they had already integrated climate change and health into their curricula. The results showed that 414 – just under 15% – of the universities had already made this integration [19]. Student-led courses existed at 12% of the universities in addition to the official curriculum [19]. Global data collection extended to other health professions was conducted in 2017-2018 by Shea et al. The results showed that 53 of the 84 (63%) responding institutions offered curricular events on climate change. Of these, 37 institutions were from the fields of public health or health sciences, 12 were from medicine, and four were from nursing [20].

One study that methodologically belongs to participatory action research investigated the integration of courses on sustainability at eight medical schools in the United Kingdom [21]. Teams of faculty and students from the participating medical schools were formed to expand existing courses or implement new courses. The teams received technical input, for example, through a seminar on planetary health education. As part of the study, the teams were supported in their subsequent project work. Curricula were revised at seven of the eight participating medical faculties. In addition to existing courses, for example, the cases for problem-based learning (POL) were expanded, lectures were supplemented to include the topic of sustainability, and the choice of an environmental focus was made possible when writing essays on the subject of global health. Implementation was not completed at the eighth college [21]. In their work, the authors emphasized the value of cross-site collaboration and the importance of bringing together individuals with different experiences and knowledge, as both approaches are conducive to inspiration and more effective dissemination of best practices.

While students were represented as part of the teams examined in the work of Walpole et al., there are also numerous primarily student initiatives found internationally that seek to integrate the topic of climate change into medical education. Accordingly, the first important statements on climate change education in medical education by medical students have been published. Three student organizations in particular are supporting the development and implementation of new courses on climate change through topic-related publications:

- In collaboration with the WHO, the International Federation of Medical Students Association (IFSMA) published a handout on climate change that explains health impacts, scientific basis, adaptation and mitigation, and policy context. This information has been prepared for the organization of student-led workshops [22].
- In Australia, a guide for sustainable and climate-friendly event design was produced by the Australian Medical Students Association (AMSA) [19].
- The Health and Environment Adaptive Response Task Force (HEART) of the Canadian Federation of Medical Students (CFMS) lists specific learning objectives in various subject areas [23].

#### 4.2. Climate-related learning objectives in medical education

In addition to student groups, lecturers and experts are also developing concrete catalogues of learning objectives. Internationally, experts on the topics of climate change and sustainability have developed a compilation of various essential and subordinate learning objectives that should be anchored in the medical curriculum at different points in time (see table 1 (Tab. 1)) [6].

A catalogue of learning objectives was developed specifically for the United Kingdom in collaboration with many experts and students [19]. This national catalogue of learning objectives was incorporated into the accreditation standards by the General Medical Council and, since 2020, obliges universities to offer teaching on sustainability as a superordinate topic [19]. Learning objectives for teaching on climate change and health can be extracted from the courses that have already taken place, expert recommendations and student evaluation. According to the Centre of Sustainable Healthcare, these can be divided into the following three areas [https://sustainablehealthcare.org.uk/priority-learning-outcomes].

1. Describe how the environment and human health interact at different levels.
2. Acquire the knowledge and skills needed for more sustainable health care systems.
3. Discuss how a physician's duty to protect and maintain human health is affected by the local and global environment.

Maxwell and Blashki took a different approach, namely, the breakdown into four distinct knowledge domains, in establishing additional topic-based learning objectives. Thus, learning objectives can be identified from the domains of factual knowledge, conceptual knowledge, skill-related knowledge, and affective knowledge (see table 2 (Tab. 2)) [4].

In particular, the importance of the first domain, namely, factual knowledge about climate change, is discussed in some studies; for example, more than 70% of Chinese medical students reported that they consider this climate-related factual knowledge to be important [12]. On the part of students and lecturers, interestingly, learning objectives that address the relationship between the environment and health are favoured. Furthermore, lecturers and students encourage the teaching of central concepts such as sustainability [24].

In addition to factual knowledge, other domains of knowledge are significant; when teaching clinical skills, instructors can take the opportunity to incorporate concepts of sustainability, for example, by reducing packaging materials and thereby reducing resource use with greenhouse gas savings [25]. Specific competencies such as the ability to communicate environmental issues to the public and contribute to sustainable management and delivery of health services are also sought after [26].

Walpole et al. found in their review of numerous publications on teaching climate-related content in medical education that knowledge of human-ecosystem relationships was highlighted as important by many authors, comparable to the aspirations of medical students [26]. Similarly, expert interviews revealed that factual knowledge is currently prioritized to fill knowledge gaps [6]. Ultimately, factual knowledge about the causes of man-made climate change is considered by some authors to be essential to sufficiently motivate health workers to act more sustainably and advocate for change [11]. In their literature review, Walpole et al. identified critical thinking as an important overarching skill that enables reflection on problems and their solutions [26].

## 5. Design options for climate-related courses and specific certificates of achievement

### 5.1. Integration of climate change content into existing courses and curricula

Medical education in many countries is currently considered to be completely overloaded with content [6]. Topics on climate change and health can either be integrated into existing curricula in various subjects, such as general medicine, paediatrics, cardiology, nephrology, psychiatry, and emergency medicine [27], or taught as a cross-cutting topic [28]. The responsibility for how climate change teaching is integrated into medical education rests with individual universities. Helpful to the development of curricular events on climate change and health are internal factors, such as the interest of the learners themselves or the relevant faculty. Support from other quarters is also perceived as valuable [20]. This help is contrasted with external factors such as political pressures, societal expectations, legislation, or technological advances [29].

Furthermore, in a study by Goldman et al., regular meetings of all disciplines involved proved to be valuable for capturing thematic overlaps and identifying common obstacles for the implementation of the curricula, as well as to improve communication in a targeted way [30].

#### 5.2. Suitable teaching methods and formats

When reviewing the methods described in the literature, it is obvious – how could it be otherwise – that the teaching methods and formats used in the field of climate teaching in medical studies can also be found in other medical didactics. For example, in a publication on climate teaching, Maxwell et al. proposed a variety of teaching methods and formats, ranging from the delivery of classical lectures to practical and project work, self-directed learning in the form of problem-based learning (PBL), and the introduction of reflective journals [4].

Internationally, the question of what advantages a climate-specific contextualization of existing teaching formats might have over separate climate teaching (through newly created courses) is being discussed; i.e., if content on climate change is embedded in existing teaching formats, such as tutorials or case discussions, this puts what is learned into context, demonstrates its relevance, and contributes to the (permanent) anchoring of the content in medical education [4]. Contextualization through case-based approaches also leads to a deepening of the learning content [29]. The argument in favour of teaching climate change separately is that students can thereby understand the connections between climate and health more comprehensively and thus learn concepts rather than facts [4].

According to many authors, the aforementioned POL teaching format seems to be particularly suitable for the topic complex of climate teaching in medical studies, regardless of whether an existing POL course series is specifically contextualized or newly established for the purpose of climate teaching [31], [32]. Thus, this teaching format seems to allow topics from the field of “eco-health” to be combined with other (medical) knowledge in a particularly organic way [32].

One possible goal of teaching climate change can be to give the participating students an impression of the diversity of this topic. In addition to knowledge and skills, attitudes and values come to the fore in the field of climate change education, which cannot be “learned” in the conventional sense. Therefore, ethical case work and discussions, group work involving diverse perspectives, and reflection exercises are particularly helpful in teaching attitudes and values [29].

The involvement of committed students takes on a special significance in the field of climate teaching, since individual medical students have a profound knowledge of climate change and its health consequences, for example, due to a previous commitment to Fridays For Future. To profitably incorporate these competencies into courses, the “flipped classroom” technique, discussion rounds and debates are particularly suitable [4]. Peer-to-peer teaching formats are also conceivable for this complex of topics [4]. The IFSMA also pursues the approach of peer education; through the handout published by the IFSMA, committed students are enabled to design workshops on the topic of climate change based on their own initiative [22].

In addition to the aforementioned teaching methods and formats, clear lesson structure, concise instructions, and approaches that demonstrate the relevance of scholarship to clinical practice also contribute to greater student engagement [21]. For example, lectures by practising physicians are perceived as valuable by students [31]. Finally, humanistic learning principles can be used to promote self-awareness and motivation in the target student population, which can lead to even more pronounced ownership and engagement [33].

#### 5.3. Certificates of achievement in the field of climate teaching

Depending on the university setting, the focus and the teaching method used (in the sense of constructive alignment), it can also be useful to provide evidence of achievement in the field of climate science [6]. Internationally, a variety of examination formats for the subject area of climate science have already been tested and established. As in many other areas of medical education, high-quality examinations should not ask for pure factual knowledge, as this would not do justice to the complexity of the topic [29]. Classical examination formats such as an MC exam seem to have limited suitability for testing transformative competencies, for example. “Deeper” knowledge could also be demonstrated and tested through student (final) presentations, reflective writing, special diaries, essays and papers, or more complex project work [29]. In the case of project work, which often binds students for an extended period of time, it is advantageous to allow students to choose their own focus [31]. In the best case, final examination guidance even contributes to the achievement of increased self-efficacy in addition to the acquisition of competencies through the completion of (smaller) transformative practice projects. Finally, it should not go unmentioned that the globally established format of “objective structured clinical examinations” (OSCE) is also suitable for the field of climate change teaching [29].

## 6. Discussion

The aim of this paper is to present the state of the art of climate science within medical education. On an international level, there are already some projects focused on the integration of this complex of topics into medical curricula. For example, learning objective catalogues and handouts for the design of specific courses exist, which are freely available and represent an important inspiration. The field of teaching climate-related content is rapidly evolving; for example, as this article goes to press, a topic-based extension of the National Competence Based Catalogue of Learning Objectives for Undergraduate Medical Education (NKLM) is already underway in Germany. Despite all the attention to detail and the dedication that medical educators sometimes display on the way to obtain the best possible course, one thing must not be forgotten: time is of the essence. Therefore, according to the authors of this review, just as important as considering the literature presented herein is the willingness to “get started”. The relative pressure of time and action, in turn, underscores the importance of an accompanying seminar evaluation to be able to make adjustments as the seminar progresses. Physicians practising today and in the future will be confronted with the health effects of climate change. Adaptation strategies must therefore be taught, as well as ways to minimize the negative ecological impacts of the health sector.

Of course, this work is also subject to certain limitations that restrict its informative value; due to its narrative character, no claim to completeness can be made. The actual knowledge of medical students about the health consequences of climate change can only be approximated. Regarding the low level of willingness of today’s medical students to act as social role models for future patients, it must be critically questioned whether this lack of willingness is due to an ignorance of the health effects of climate change or whether this responsibility is seen to lie with other professions. Few teaching projects seem to have been developed or scientifically described within human medicine thus far; therefore, studies from neighbouring disciplines are often used. This makes it difficult to make a statement specifically related to medical students. Ultimately, however, teaching on the links between climate change and health seems to have depended heavily on the initiative of individual student groups, lecturers, faculties or universities. It is therefore necessary to expand the medical curriculum to include content on climate change on a mandatory and comprehensive basis to take the importance of this topic into account.

## 7. Further information

- The Sustainable Healthcare Education Network (SHE) of the Centre of Sustainable Healthcare (CSH Networks) provides a peer network to support teaching about sustainable health care. CSH Networks: https://networks.sustainablehealthcare.org.uk/
- The Planetary Health Report Card Initiative https://phreportcard.org/ is a student-led initiative to encourage universities and faculties to become more involved in teaching about climate change. It features an interactive map of teaching opportunities, currently particularly in the U.S. and England.
- The Canadian Federation of Medical Students (CFMS) has published a catalogue of learning objectives with recommendations on which topics to cover on climate change and into which existing subjects they can be integrated: https://www.cfms.org/what-we-do/global-health/heart-competencies.

## Funding

Funded by the Baden-Württemberg Stiftung.

## Competing interests

The authors declare that they have no competing interests.

## Figures and Tables

**Table 1:**
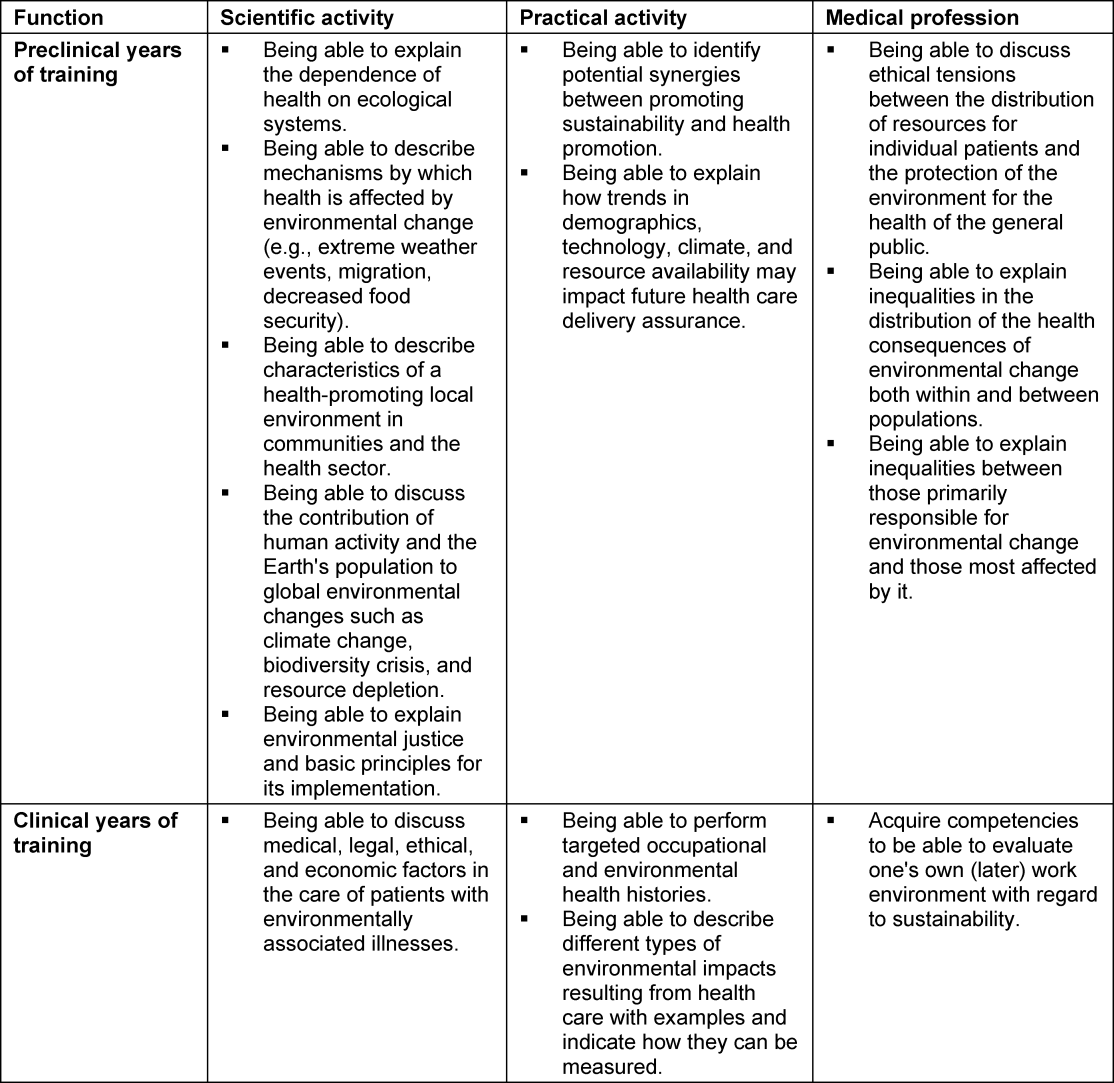
Learning objectives in curriculum sections, modified after Teherani A. et al., 2017 [6]

**Table 2:**
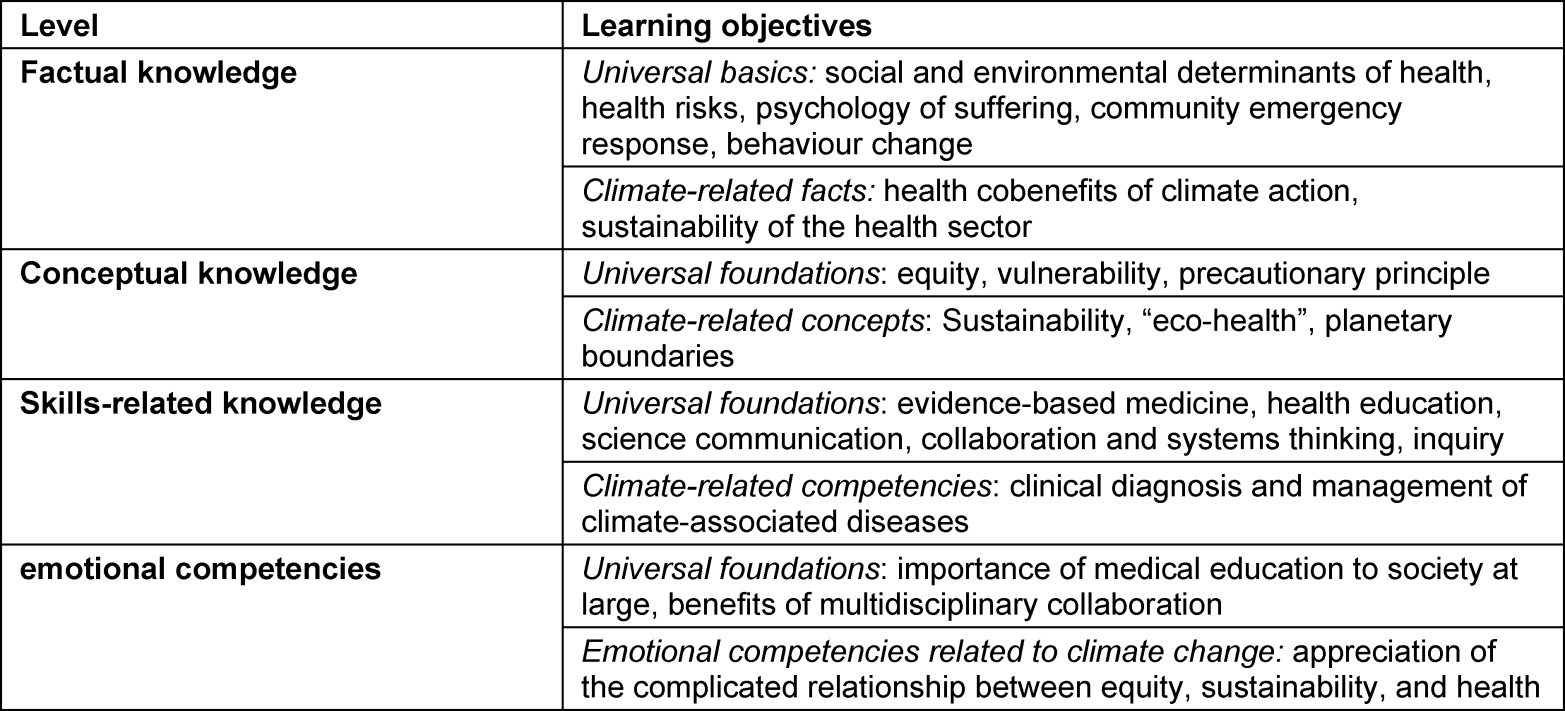
Learning objectives for four knowledge domains (adapted from: Maxwell J, Blashki G 2016 [4])
